# A study on the temperature profile of bifurcation tunnel fire under natural ventilation

**DOI:** 10.1371/journal.pone.0262546

**Published:** 2022-01-10

**Authors:** Jianlong Zhao, Yanfeng Li, Junmei Li, Jiaxin Li

**Affiliations:** Beijing Key Laboratory of Green Built Environment and Energy Efficient Technology, Beijing University of Technology, Beijing, China; Shahrood University of Technology, ISLAMIC REPUBLIC OF IRAN

## Abstract

This study simulated a series of bifurcation tunnel fire scenarios using the numerical code to investigate the temperature profile of bifurcation tunnel fire under natural ventilation. The bifurcation tunnel fire scenarios considered three bifurcation angles (30°, 45°, and 60°) and six heat release rates (HRRs) (5, 10, 15, 20, 25, and 30 MW). According to the simulation results, the temperature profile with various HRRs and bifurcation angles was described. Furthermore, the effects of bifurcation angles and HRRs on the maximum temperature under the bifurcation tunnel ceiling and the temperature decay along the longitudinal direction of the branch were investigated. According to the theoretical analysis, two prediction models were proposed. These models can predict a bifurcation tunnel fire’s maximum temperature and longitudinal temperature decay in the branch. The results of this study could be valuable for modelling a bifurcation tunnel fire and benefit the fire engineering design of bifurcation tunnels.

## 1. Introduction

In recent years, improvements to urban underground transportation networks have led to the increased complexity of tunnels [1, 2]. These complex tunnels may lead to more extraordinary tunnel fire incidents [3] that could cause substantial finical loss and heavy casualties [4]. One of these complex tunnels is the bifurcation tunnel that consists of the main tunnel and a branch. Due to the different fire dynamics in bifurcation tunnels compared to a traditional single tunnel, conventional fire prevention measures do not apply to bifurcation tunnels [5, 6]. Therefore, it is necessary to study the dynamics of bifurcation tunnel fires.

In practical projects, the tunnel structure material is mostly concrete, and the concrete strength decreases when the environmental temperature is higher than 300°C [7]. For this reason, the temperature characteristics of a tunnel fire, such as maximum temperature and temperature decay, are essential parameters and have been studied for decades. For example, Alpert [8] and Kurioka et al. [9] developed maximum temperature prediction models for a tunnel fire. Moreover, researchers have carried out studies on the factors affecting the maximum temperature, such as the location and number of fire sources [10–12], the shape of tunnel cross-section [13, 14], velocity of longitudinal ventilation [15], vehicle blockage in a tunnel [16, 17], ambient pressure [18, 19], and the slope of the vertical shaft [20]. In addition to the maximum temperature, researchers have also investigated temperature decay. Evers et al. [21] proposed a well-known exponential equation to predict temperature decay. Hu et al. [22] conducted full-scale burning experiments and developed an exponential model to describe temperature decay. Gao et al. [23] investigated a tunnel fire’s longitudinal and transverse temperature distribution by model-scale experiments. Lastly, Ye et al. [24] conducted experiments to study the longitudinal temperature decay of a utility tunnel fire.

The studies mentioned above focused on traditional signal tunnels, but there may be bifurcation tunnels in real-world situations. The bifurcation tunnel has a specific construction that can influence the spread of fire smoke and lead to a bifurcation tunnel fire significantly different from traditional signal tunnel fire. Hence, some researchers have studied the bifurcation tunnel fires. First, Liu et al. [25] conducted full-scale fire experiments and investigated the temperature distribution in a bifurcation tunnel node area. Next, Liu et al. [26] carried out model-scale experiments and full-scale simulations to study the influence of metro tunnel inclination on tunnel fire smoke temperature under natural ventilation. Liu et al. [27] also discussed the critical velocity that prevents smoke from spreading into the cross-passage between two metro tunnels. Then, according to fire tests in a bifurcation tunnel model, Huang et al. [28] proposed prediction equations of maximum temperature and critical velocity. After then, Chen et al. [29] experimentally investigated the influence of branches on the smoke spread of a bifurcation tunnel fire. Besides, Chen et al. [30, 31] assessed the effects of the confined ratio and ramp slope of branch tunnels on the behaviours of a bifurcation tunnel fire by small-scale experiments. Finally, Lei et al. [32] investigated the effect of fire locations on the temperature distribution of a bifurcation tunnel fire through small-scale experiments. Furthermore, because the fire may occur at any location in the bifurcation tunnel, the effects of different transverse and longitudinal fire source locations on the temperature characteristics of bifurcation tunnel fires have also been investigated [33, 34].

In summary, several earlier studies were carried out to examine the temperature profiles of bifurcation tunnel fires. However, the coupling effect of bifurcation angles and HRRs on the temperature profile distribution of bifurcation tunnel fires seems to be comparatively little studied. Consequently, this study conducted a series of full-scale simulations using the numerical code to investigate the temperature profile characteristics of bifurcation tunnel fires under natural ventilation. According to the theoretical analysis of the simulation results, two equations were proposed that considered the effects of bifurcation angles and HRRs. These equations can predict the maximum temperature of bifurcation tunnel fire and the temperature decay along the longitudinal direction of the branch, respectively.

## 2. FDS simulation setup

### 2.1. Model design

**Fig 1:**
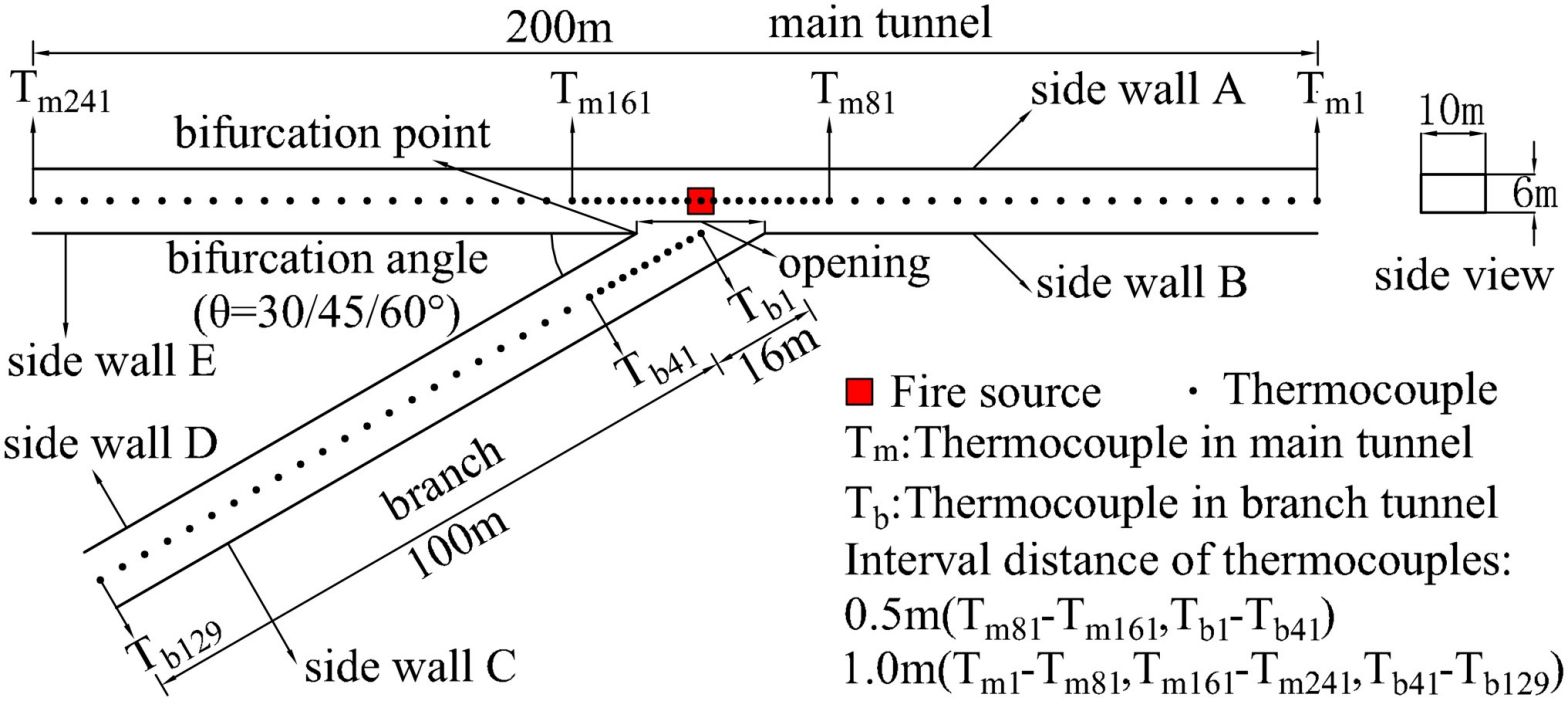
Schematic diagram of bifurcation tunnel geometric model.

As shown in Fig 1, a full-scale bifurcation tunnel was constructed using Fire Dynamics Simulator (FDS) code (Version: 6.7.1) according to a reduced-scale tunnel [29]. The bifurcation tunnel consisted of a main tunnel and a branch. The main tunnel and branch lengths were 200m and 100m, respectively. The tunnel’s cross-section was rectangular, with a height of 6m and a width of 10m. The bifurcation angle between the main tunnel and the branch was 30°, 45°, and 60°, as defined in Fig 1. Previous studies [26, 30–32, 34] have used the bifurcation angles of 30° and 45°.

The tunnel structure material was set as “concrete,” Table 1 shows the concrete properties. Three portals of the bifurcation tunnel were set as “open.” The ambient temperature and pressure were set as 20°C and 101 kPa, respectively. The fire source was located in the middle of the main tunnel, and polyurethane was selected as the fuel [35]. The dimensions of the fire source were 2.0 m × 2.0 m, and the HRRs of the fire source were set as 5, 10, 15, 20, 25, and 30 MW according to previous studies [36–38].

**Table 1:** Thermal properties of concrete.

Name	Specific heat	Conductivity	Density	Emissivity	Absorption coefficient
Unit	kJ / (kg K)	W (m K)	kg / m^3^	-	l / m
Value	1.04	1.80	2280	0.90	50000

Thermocouples measured the smoke temperature under the bifurcation tunnel ceiling. The thermocouples thickness was 1.0 mm, and the distance between thermocouples and the tunnel ceiling was 0.1 m. In the branch and the main tunnel, the thermocouples labelled T_b1_-T_b129_ and T_m1_-T_m241_, respectively. As shown in Fig 1, the interval of the thermocouples near the fire source (T_m81_-T_m161_, T_b1_-T_b41_) and at other places (T_m1_-T_m81_, T_m161_-T_m241_, T_b41_-T_b129_) was set as 0.5 m and 1.0 m, respectively.

### 2.2. Sensitivity study of grid size

Grid size is an important parameter for FDS simulations. For example, the FDS user guide [39] indicates that in simulations involving buoyant plumes, how well the flow field resolved is reflected by a non-dimensional expression D*/*δ*_*x*_. Among the above expression, *δ*_*x*_ is the nominal size of a mesh cell, and D* is the characteristic fire diameter that the following equation can calculate:

$$D^{*}={\left(\frac{Q}{\rho_{\infty }{\text{c}}_{p}T_{\infty }\sqrt{g}}\right)}^{\frac{2}{5}}$$ (1)

where *Q* is the heat release rate of the fire source, *T*_∞_ is the environment temperature, *g* is gravity acceleration, *ρ*_∞_ is the environment air density, and *c*_*p*_ is the constant pressure-specific heat capacity.

**Fig 2:**
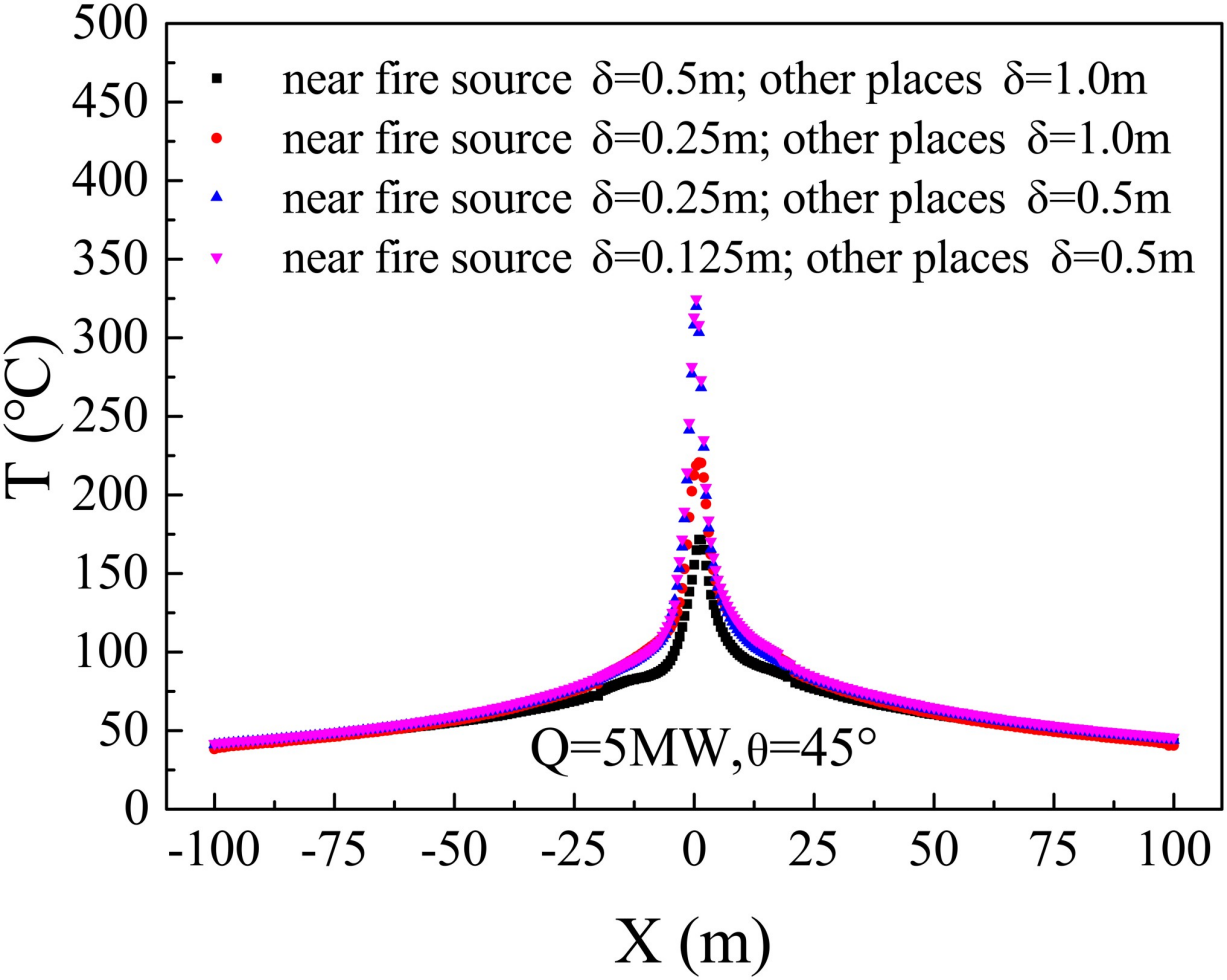
Grid size sensitivity analysis.

The accuracy of simulation results improves with D*/*δ*_*x*_ decreases. Meanwhile, the smaller the grid size is, the more accurate the simulation results are, and a longer calculation time is needed. Therefore, a grid sensitivity study was conducted to select an appropriate gird size with the most accurate simulation results in the shortest calculation time. According to the above expression, the minimal heat release rate determined the appropriate grid size. Then four grid sizes for Q = 5 MW, θ = 45° were tested in this study, and a typical comparison was shown in Fig 2. It shows that the maximum temperature near the fire source increases with a smaller grid size, while the temperature distribution at other places shows no significant differences. Thus, the grid size near the fire source and other places was set as 0.25 m and 0.50 m, respectively. The region near the fire source was within 20 m from the fire source in the main tunnel (T_m81_-T_m161_) and the opening central in the branch (T_b1_-T_b41_), as shown in Fig 1. Several numerical simulation studies have adopted a similar grid system [34, 40–42].

### 2.3. Validation of the numerical tool

According to the Froude scaling law, Chen et al. [29] built a 1:10 bifurcation tunnel model with a concrete precast lab. The main tunnel and branch length was 10 m and 5 m, respectively. The tunnel’s cross-section was rectangular, which dimensions were 0.6 m (height) and 1.0 m (width). The bifurcation angle between the main tunnel and branch was 45°. They conducted experiments in this scale tunnel model to investigate the smoke characteristic of bifurcation tunnel fires. For experiment 49 # in Chen’s study, the heat release rate of a model scale was 16 kW, and the corresponding fire size for the full-scale model was 5 MW based on the Froude scaling law. To validate that FDS can offer reliable predictions for bifurcation tunnel fires, we compared the results of the experiment and simulation that the heat release rate was 5 MW, and the bifurcation angle was 45°.

**Fig 3:**
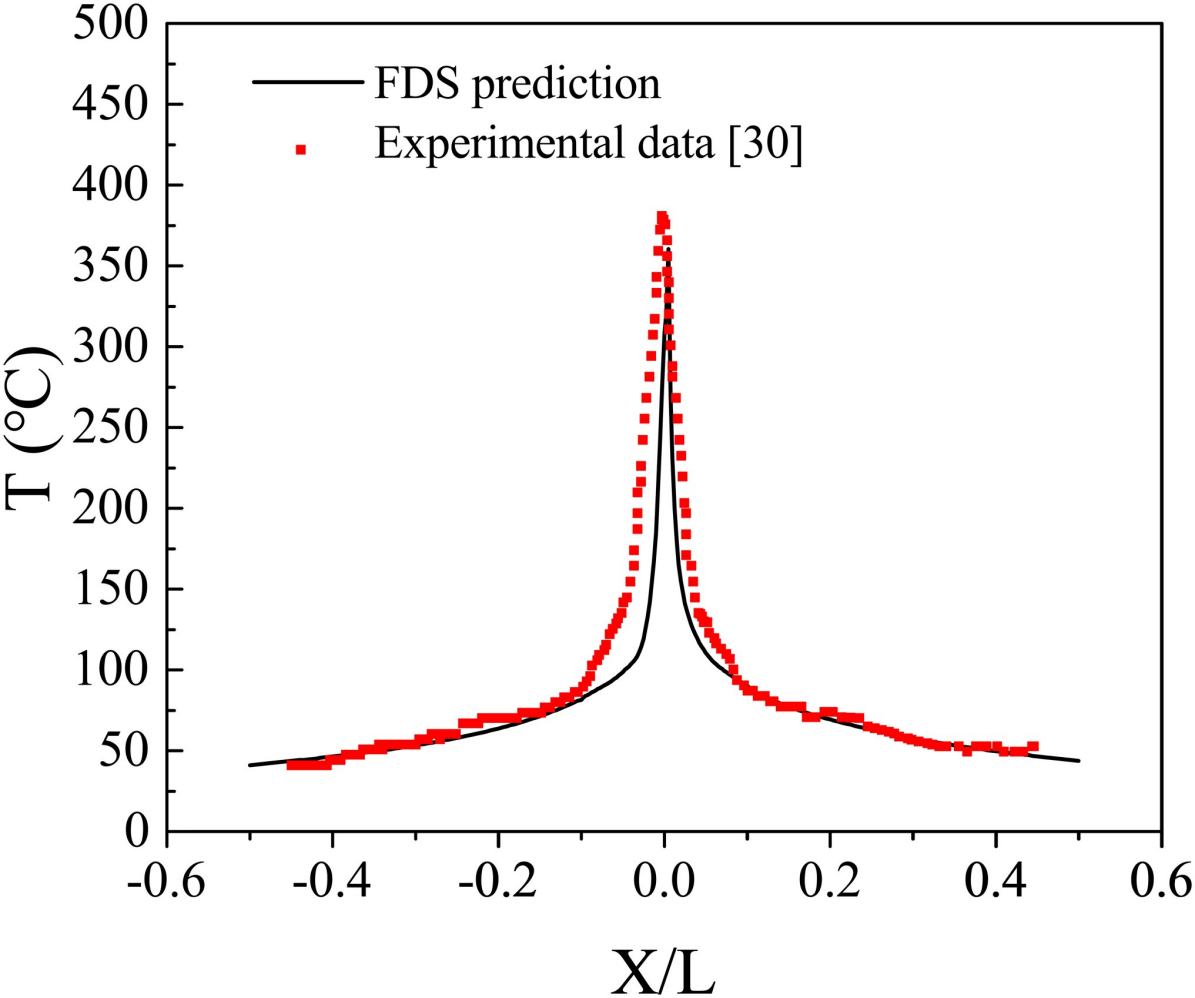
Comparison between FDS prediction and experimental data.

Fig 3 shows the experimental data of Chen et al. [29] and the prediction of FDS in this study. *T* is the temperature at different positions along the longitudinal direction of the main tunnel. *X/L* is the ratio between the distance at the fire source and the length of the main tunnel. The FDS prediction of the current study and Chen’s experimental data have a similar tendency, which means that the simulation and experiment results match well. However, the temperature values between the simulation and experimental data have some differences, especially near the fire source. These differences might be due to some inconsistencies between the simulation and experimental conditions; therefore, a reasonable deviation exists between the simulation and experimental results.

### 2.4. Data selection

**Fig 4:**
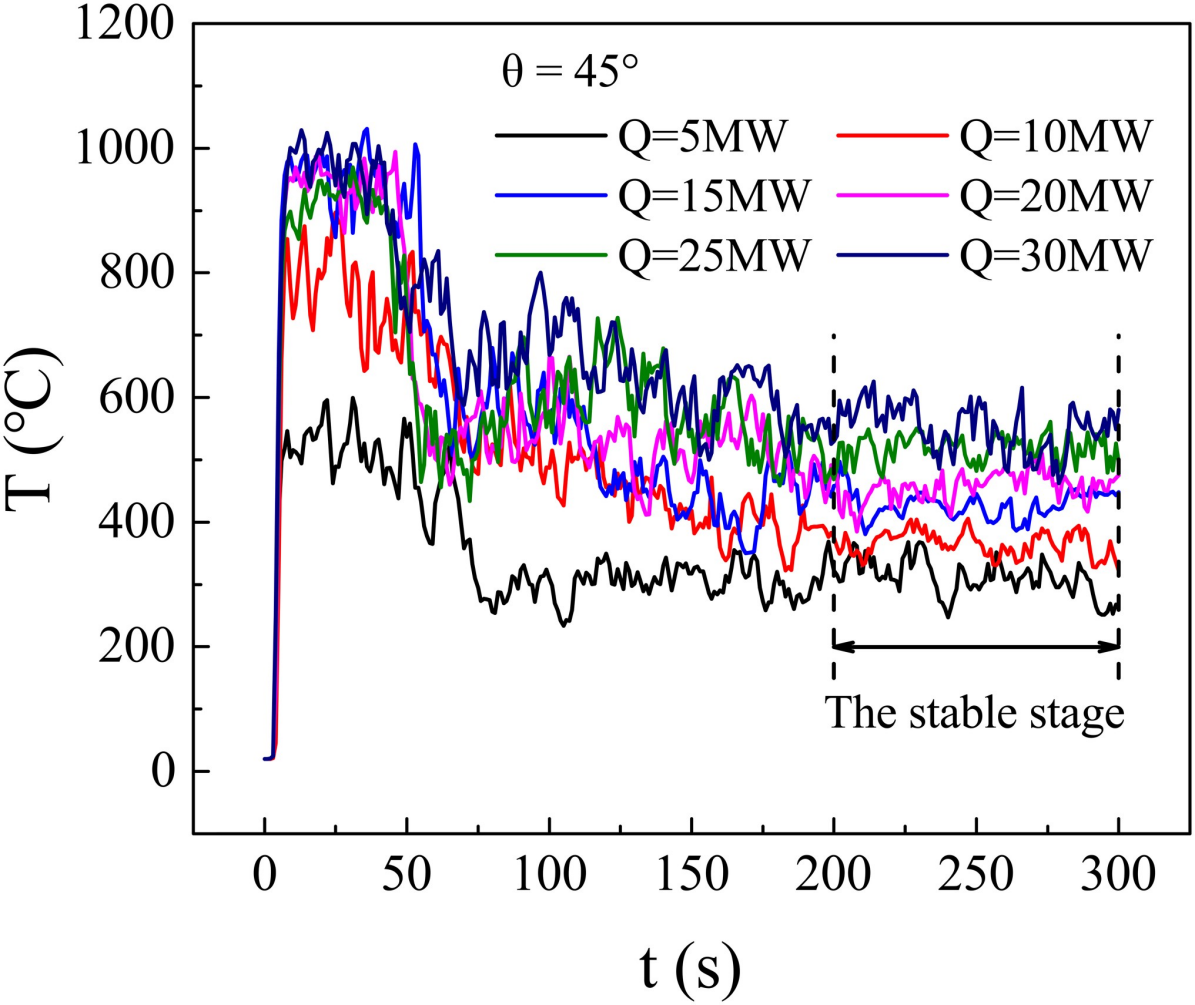
Temperature-time curves for various heat release rates at bifurcation angle of 45°.

In this paper, the simulation time was 300s, and the data at the stable stage was selected to analyse the temperature profile under the bifurcation tunnel ceiling. Fig 4 shows the smoke temperature variations above the fire source for various heat release rates at the bifurcation angle of 45°. It indicates that the smoke temperature first increases, then gradually descends, and finally reaches a stable state for each heat release rate. The time required for the smoke temperature to reach a stable state increases with the heat release rate, and it reaches a stable stage after 200s for various heat release rates. Therefore, the data between 200 s and 300 s were extracted for analysis.

## 3. Results and discussion

### 3.1. Temperature and velocity contours under the ceiling of a bifurcation tunnel

**Fig 5:**
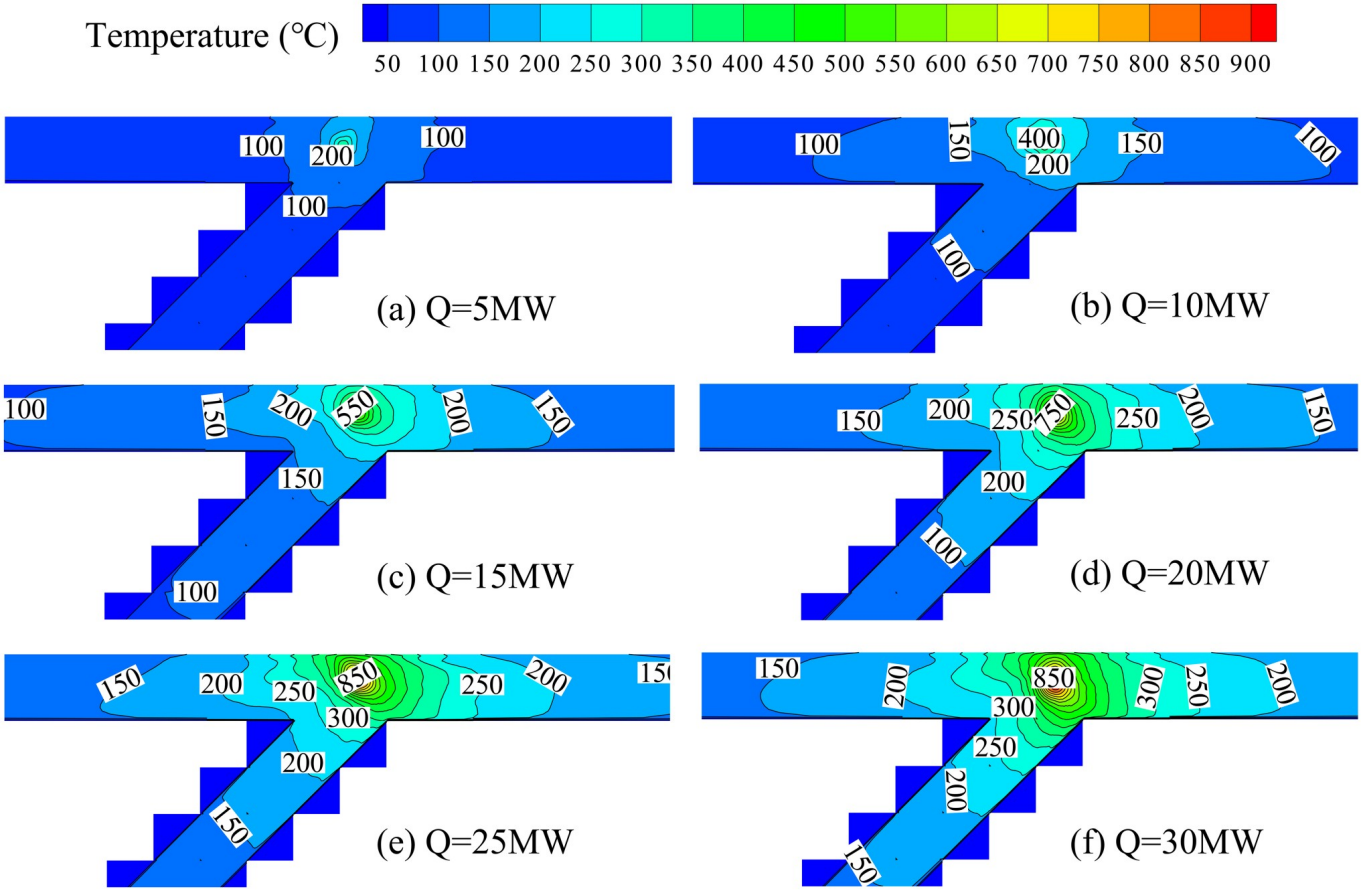
Temperature contours for various HRR at bifurcation angle of 45° (top view).

Fig 5 shows the temperature contours for various HRRs at a bifurcation angle of 45°. The thin black lines in the contours are isotherms, and the numbers around these lines are temperature values corresponding to the isotherms. In the following paragraph, take the isotherm with a temperature of 200°C as an example to describe the temperature profile under the bifurcation tunnel ceiling with the heat release rate increases.

The evolution of the temperature contour with increasing HRR can be divided into four stages according to the relative position of the bifurcation tunnel sidewall and isotherm. Firstly, when HRR is 5 MW, the sidewall of the bifurcation tunnel has no limitation to the isotherm, and the isotherm concentrates at the middle of the main tunnel ceiling. Secondly, when the HRR increases to 10 MW, the isotherm is limited by sidewall A, and it longitudinally expands upstream and downstream of the main tunnel along this sidewall simultaneously. Thirdly, when the HRR continues to increase, the isotherm is limited by sidewalls B and C (Q = 15 MW), and it continually expands (Q = 20 MW) until it reaches the bifurcation point (Q = 25 MW). Finally, the isotherm is also limited by sidewalls D and E. Then, the isotherm occupies the entire ceiling of the bifurcation tunnel (Q = 30 MW) and longitudinally expands along the main tunnel and the branch.

Fig 5 also shows that the maximum temperature position of bifurcation tunnel fire inclines upstream of the main tunnel, which is significantly different from a traditional single tunnel. For a traditional single tunnel, the fire plume symmetrically entrains fresh air from two tunnel portals. Thus, the temperature under the ceiling is also symmetrically distributed around the fire plume, and the maximum temperature position under the tunnel ceiling is above the fire source. However, the fire plume can entrain fresh air from the branch, upstream and downstream of the main tunnel for a bifurcation tunnel. The fresh air entrained from upstream of the main tunnel is less than that entrained from the branch and downstream of the main tunnel. The plume temperature decreases with increased fresh air entrained by the plume. Therefore, the smoke temperature upstream of the main tunnel is higher than that in the branch and downstream of the main tunnel. Then, the maximum temperature position under the bifurcation tunnel ceiling will appear upstream of the main tunnel.

**Fig 6:**
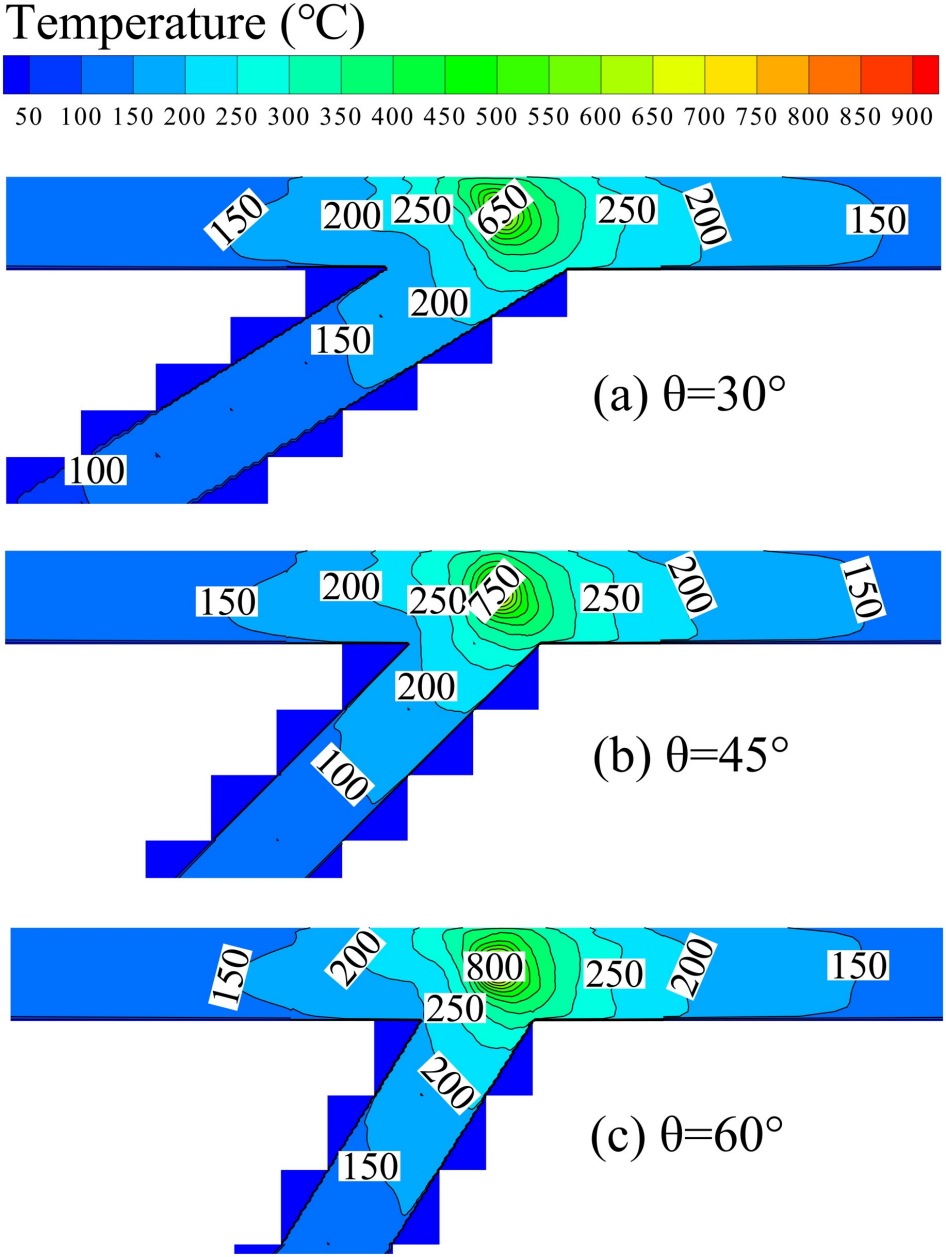
Temperature contours for various bifurcation angles at HRR of 20 MW (top view).

**Fig 7:**
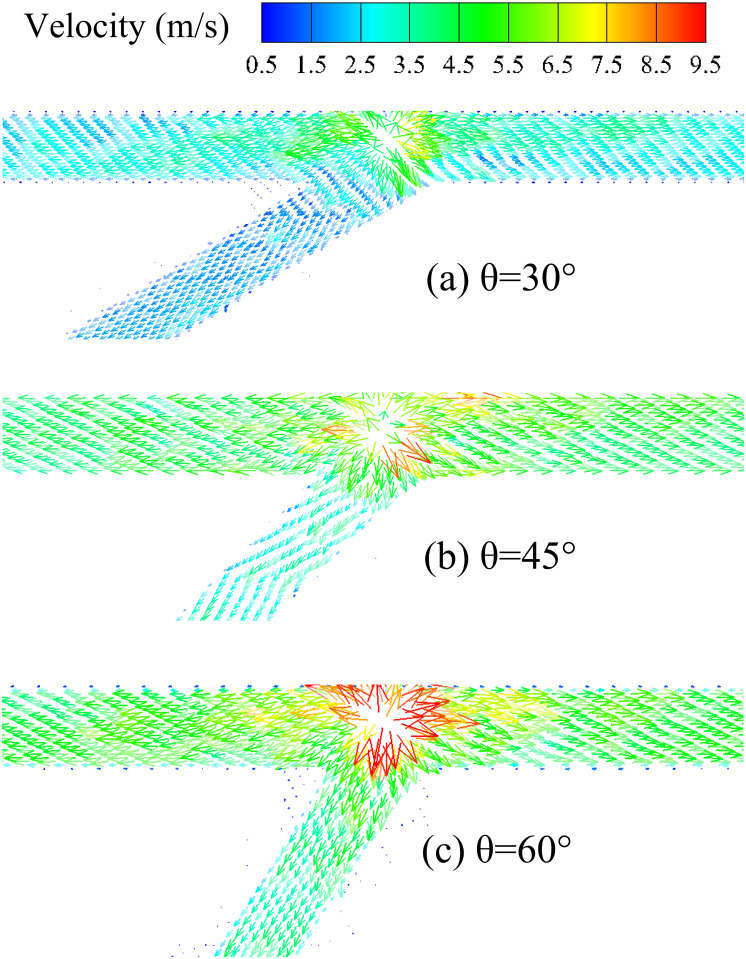
Velocity contours for various bifurcation angles at HRR of 20 MW (top view).

This study also investigated the influence of the bifurcation angle on the maximum temperature of bifurcation tunnel fires. Figs 6 and 7 are temperature and velocity contours for various bifurcation angles at an HRR of 20 MW, respectively. The maximum temperature under the bifurcation tunnel ceiling increases with the bifurcation angle (as shown in Fig 6) because the velocity of the ceiling-jet spreading towards the branch portal increases with the bifurcation angle(as shown in Fig 7). Then, the ceiling-jet will prevent the fresh air flow into the main tunnel and lead to maximum temperature increases.

### 3.2. Maximum temperature under the ceiling of bifurcation tunnel

Previous studies [43, 44] have indicated that the maximum temperature of tunnel fire, Δ*T*_max_, is related to the heat release rate *Q*, the environment air density *ρ*_∞_, the constant pressure-specific heat capacity of environment air *c*_*p*_, the environment temperature *T*_∞_, the gravitational acceleration *g*, and the tunnel height *H*. For this work, the dimensionless bifurcation angle *α* (*πθ*/180°) also influences the maximum temperature. Therefore, the following equation can express the maximum temperature of a bifurcation tunnel fire:

$$\Delta T_{\text{max}}=f\left(Q,\rho_{\infty },c_{p},T_{\infty },g,H,\alpha \right)$$ (2)

Based on dimensional analysis, Eq (2) can be further reduced to the following equation:

$$\frac{\Delta T_{\text{max}}}{T_{\infty }}=f\left(\frac{Q}{c_{p}\rho_{\infty }T_{\infty }g^{1/2}H^{5/2}},\alpha \right)$$ (3)

where,

$$Q^{*}=\frac{Q}{c_{p}\rho_{\infty }T_{\infty }g^{1/2}H^{5/2}}$$ (4)

Q* is the dimensionless HRR that describe the relative magnitudes of fires. Then, the following equation can be obtained by combining Eqs (3) and (4):

$$\frac{\Delta T_{\text{max}}}{T_{\infty }}=f\left(Q^{*},\alpha \right)$$ (5)

The dimensionless maximum temperature (Δ*T*_*max*_/*T*_∞_) against the dimensionless HRR (Q*) is shown in Fig 7, which indicates that Δ*T*_*max*_/*T*_∞_ increases linearly with Q*. Hence, it is assumed that the fitted equation which can correlate Δ*T*_*max*_/*T*_∞_ and Q* is as follows:

$$\frac{\Delta T_{max}}{T_{\infty }}=m+nQ^{*}$$ (6)

where *m* and *n* are the fitting coefficients related to the bifurcation angle.

**Fig 8:**
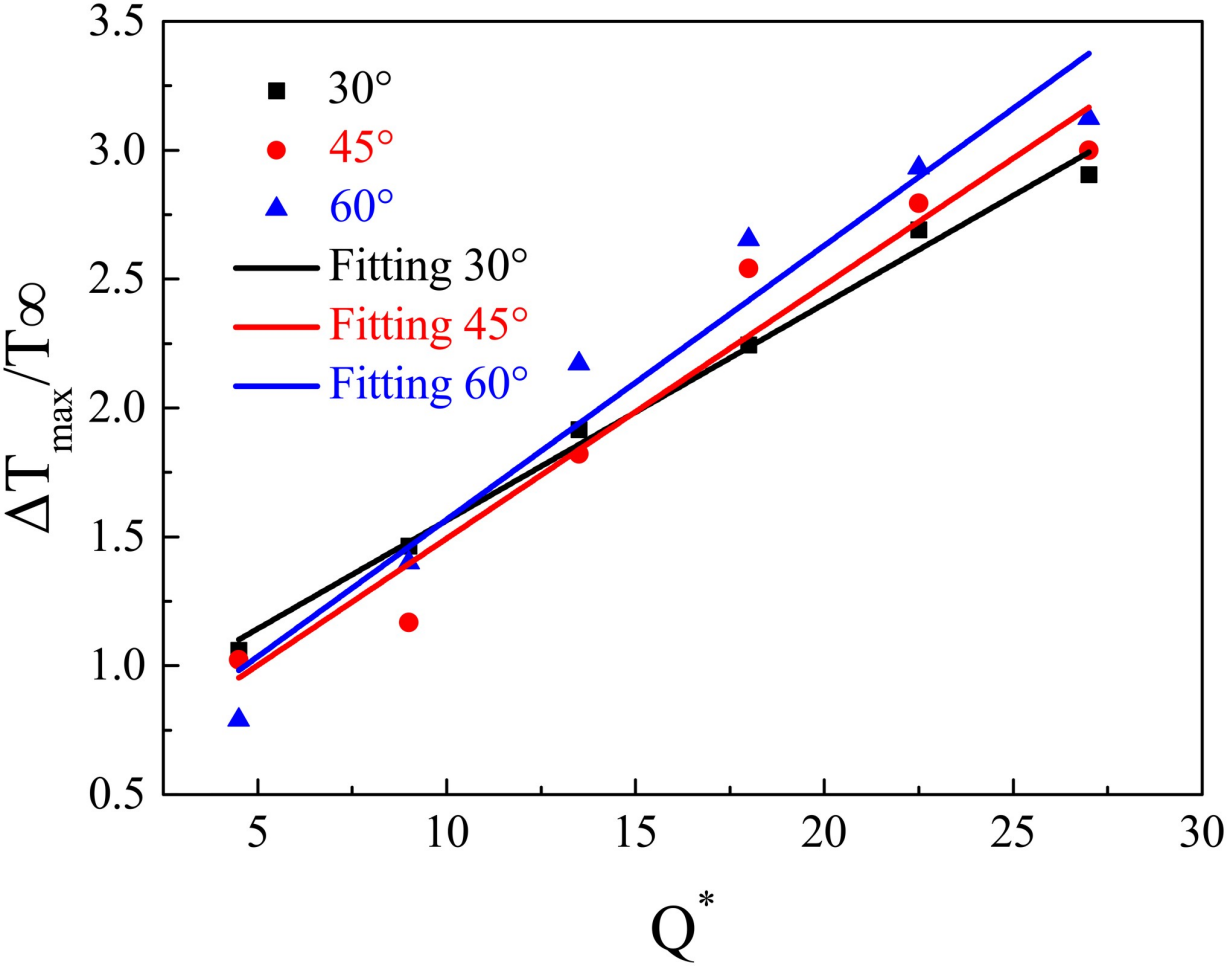
Δ*T*_*max*_/*T*_∞_ against Q* for various bifurcation angles.

Fig 8 shows for various bifurcation angles (30°, 45°, 60°), although Δ*T*_*max*_/*T*_∞_ against Q* has the same trend, there are also some differences because the coefficients *m* and *n* varied with the bifurcation angles. Table 2 lists the fitted values of *m* and *n* with different bifurcation angles.

**Table 2:** The fitted values of coefficients *m* and *n*.

*α*	0.523	0.785	1.047
*m*	0.723	0.590	0.505
*n*	0.084	0.098	0.106

**Fig 9:**
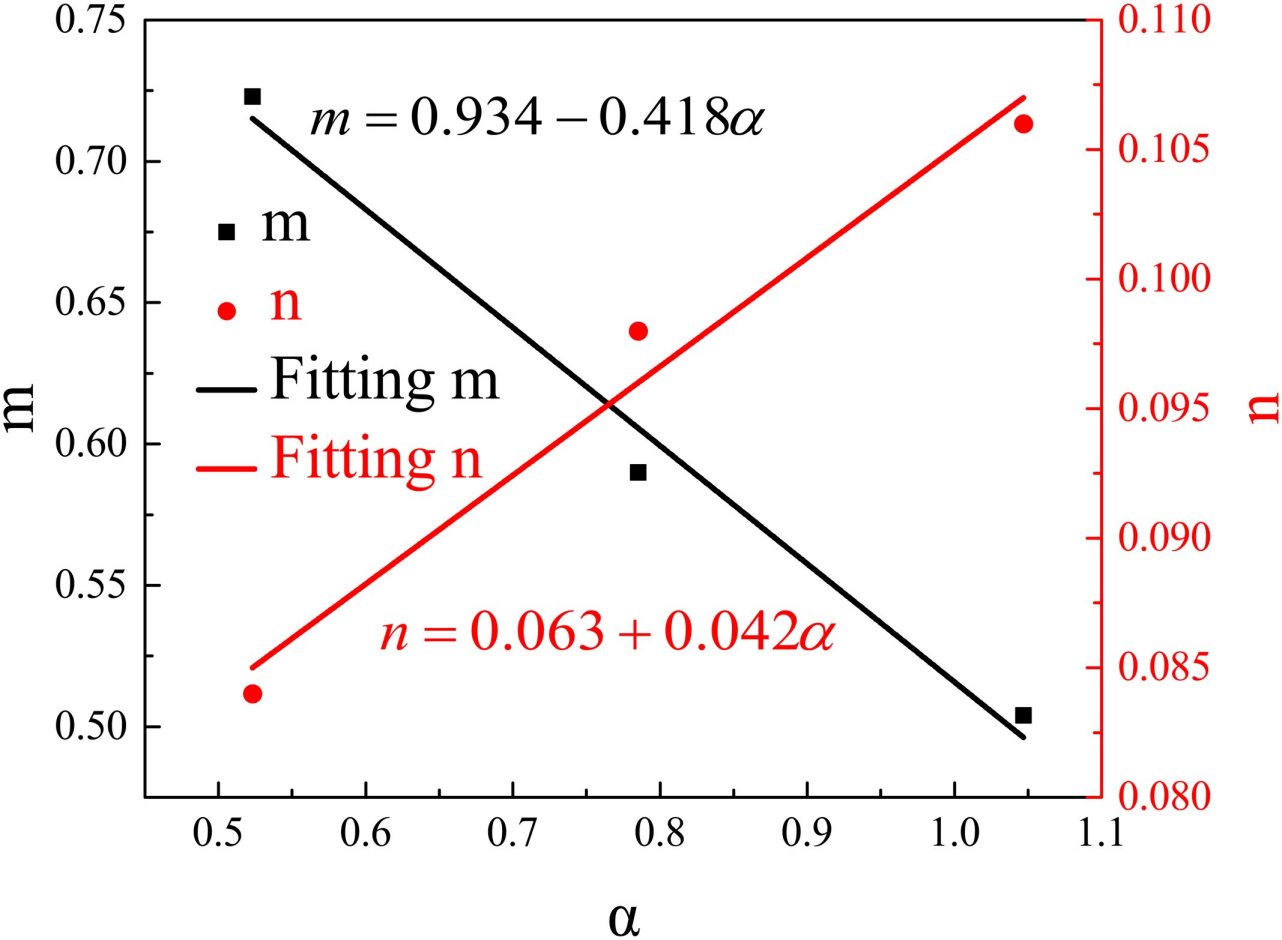
Fitting coefficients of m and n against bifurcation angles.

Fig 9 plots *m* and *n* against the bifurcation angles. The *m* and *n* values decrease and increase linearly with the bifurcation angle, respectively. Therefore, the data in Fig 9 can correlate by liner functions, and the fitted equations are as follows:

m=0.934−0.418α (7)

n=0.063+0.042α (8)

Finally, the following equation can be obtained by incorporating Eqs (7) and (8) into Eq (6).


$$\frac{\Delta T_{max}}{T_{\infty }}=(0.934-0.418\alpha )+(0.063+0.042\alpha )Q^{*}$$ (9)

This equation simultaneously considers the effects of the bifurcation angle and HRR on the maximum temperature of a bifurcation tunnel fire.

**Fig 10:**
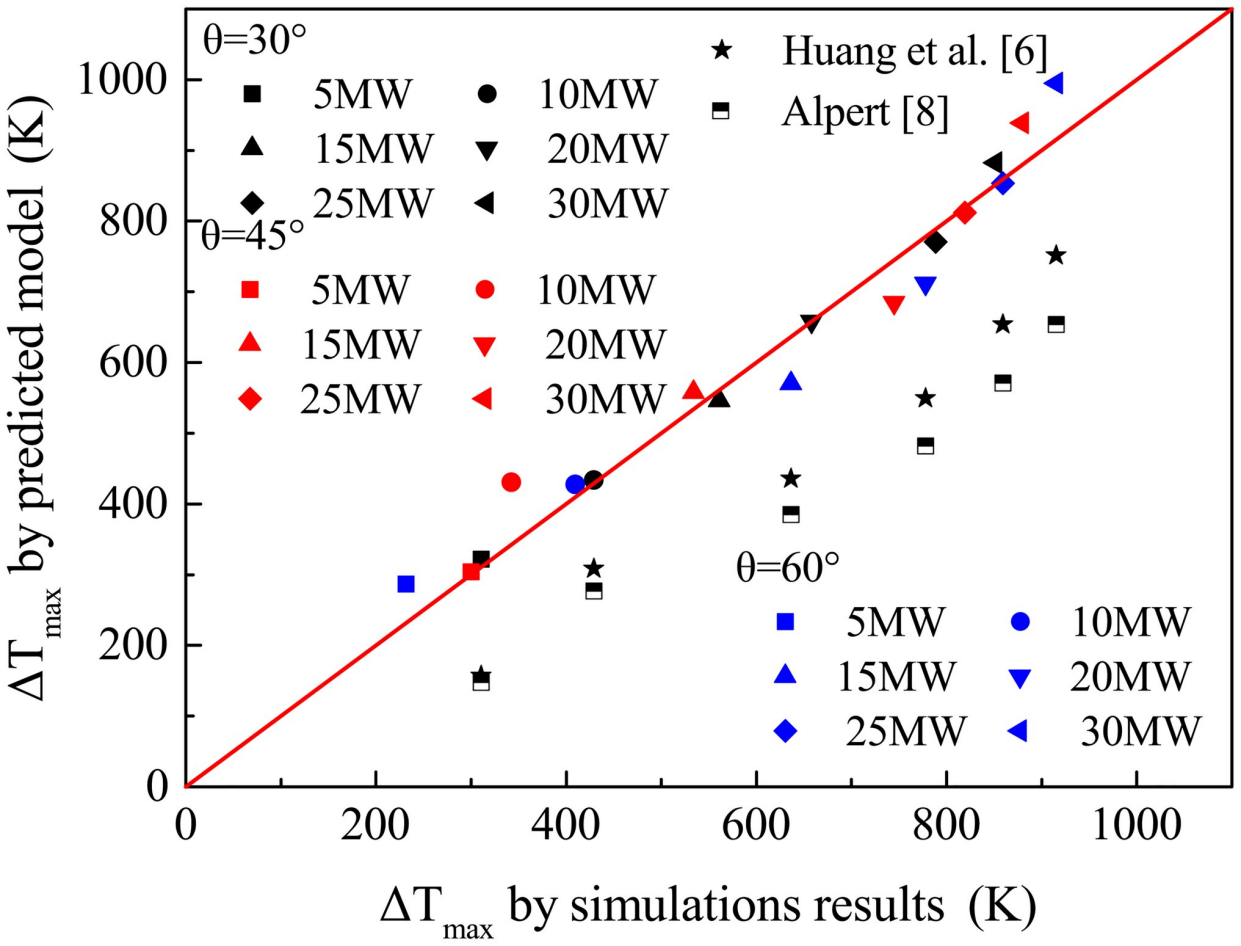
Δ*T*_*max*_ obtained by simulations and calculated by models.

Fig 10 shows the calculated values by Eq (9), the predicted values of models derived from Alpert [8] and Huang et al. [6], and data of simulation. There are insignificant differences between the values calculated by Eq (9) and the simulation results. Therefore, Eq (9) can predict the maximum temperature of a bifurcation tunnel fire. However, Alpert’s and Huang’s models both underestimate the maximum temperature of the bifurcation tunnel fire scenarios studied in this study. The reason is that Alpert’s model was derived from traditional single tunnel fire tests and did not consider the effect of bifurcation angle on maximum temperature. Therefore, a significant deviation existed when adopting Alpert’s model to predict the maximum temperature of a bifurcation tunnel fire. On the other hand, although Huang et al. derived their model from bifurcation tunnel fire experiments, the bifurcation angles they focused on were 5°, 10°, and 15°, which were much smaller than those of this study. As a result, Huang’s model cannot be directly applied to this work.

### 3.3. Effect of bifurcation angle on longitudinal temperature decay in branch

In the following paragraphs, we will explore the effect of the bifurcation angle on temperature decay along the longitudinal direction of the branch. Previous studies [43–46] have suggested that the longitudinal temperature decay of a traditional single tunnel fire follows a double exponential law. Therefore, the following equation can describe the longitudinal temperature distribution in the branch:

$$\frac{\Delta T_{\text{r}}}{\Delta T_{op}}=\chi e^{\gamma \left(\frac{r}{H}\right)}+(1-\chi )e^{\eta \left(\frac{r}{H}\right)}$$ (10)

where *r* is the distance from branch tunnel opening, Δ*T*_r_ is the temperature rise away r meters from branch tunnel opening, Δ*T*_*op*_ is the temperature rise at the branch tunnel opening, *χ*, *γ* and *η* are fitting coefficients, they should be related to bifurcation angle and HRR.

**Fig 11:**
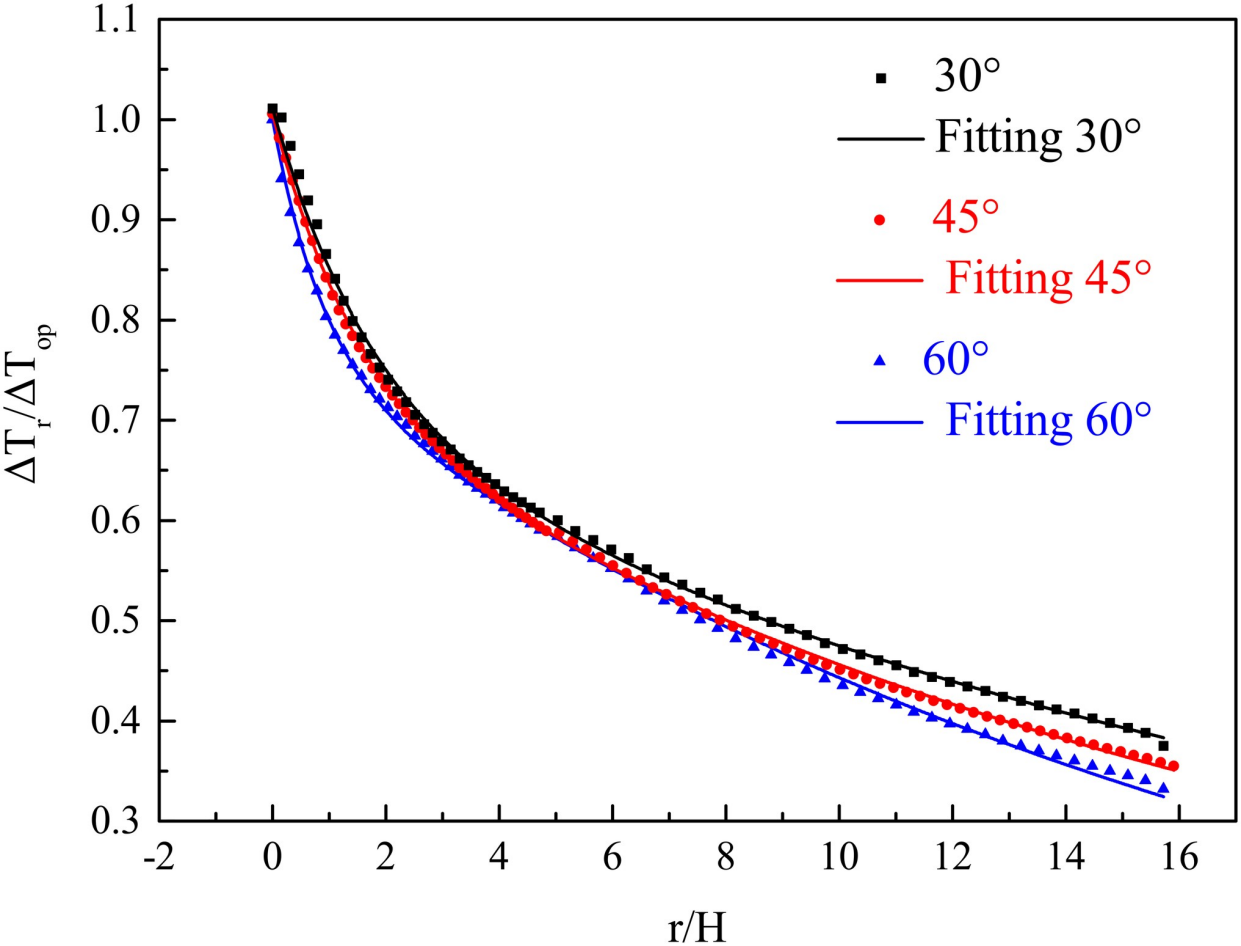
Dimensionless temperature rise (Δ*T*_r_/Δ*T*_*op*_) against dimensionless distance (*r*/*H*) along longitudinal direction of branch tunnel with HRR of 5 MW.

Fig 11 shows the fitting results using Eq (10) and the simulation data, which indicates that the dimensionless temperature rise decreases more slowly when the bifurcation angle increases. Table 3 lists the fitted coefficient values in Eq (10). Analysing these values shows that *χ* can be considered as a constant 0.282, while *γ* and *η* decrease linearly against the bifurcation angle.

**Table 3:** The fitted values of coefficients, *χ*, *γ* and *η*.

*α*	0.523	0.785	1.047
*χ*	0.286	0.282	0.279
*γ*	-0.573	-0.887	-1.136
*η*	-0.003	-0.004	-0.005

**Fig 12:**
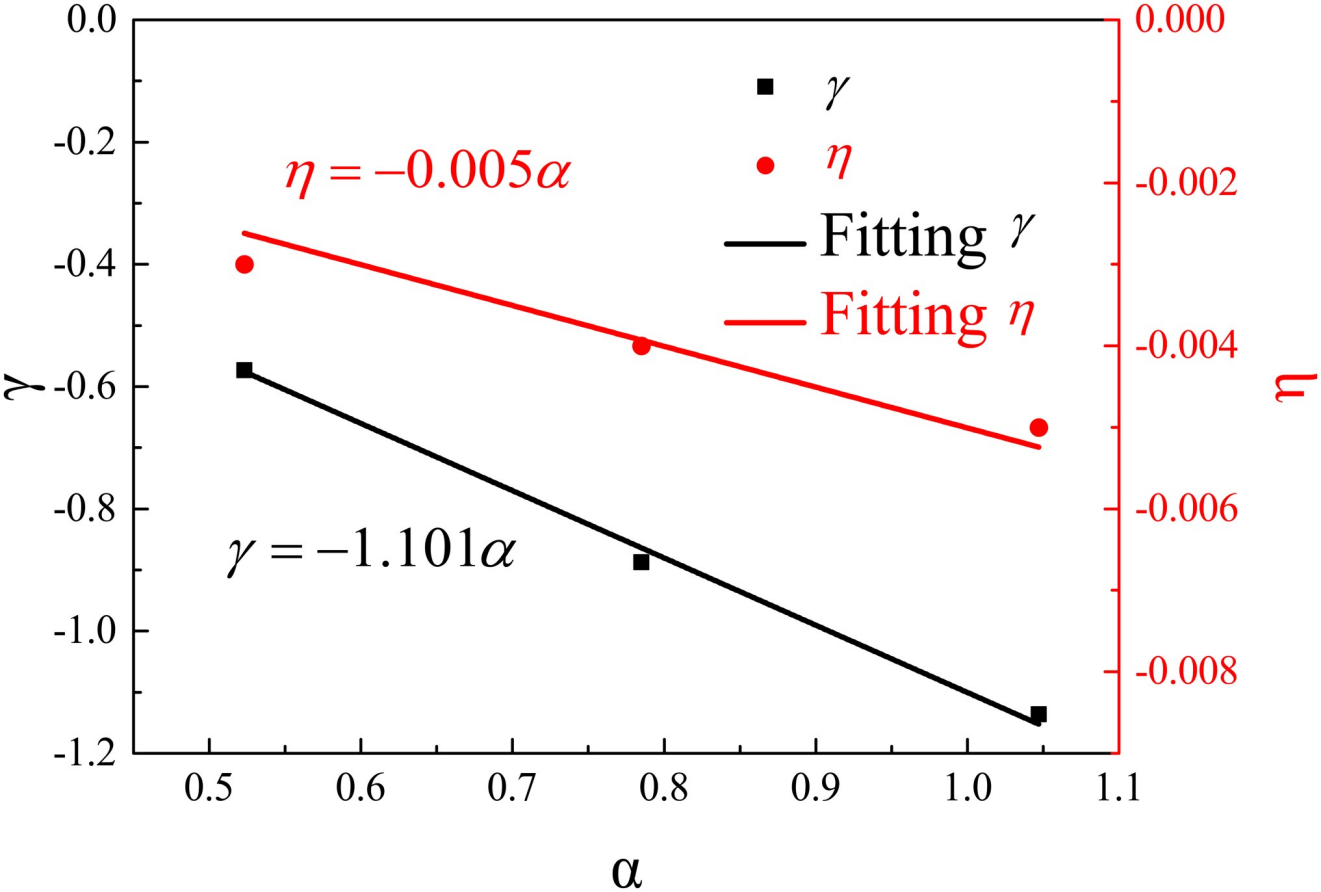
Fitting coefficients of *γ* and *η* against bifurcation angle.

Fig 12 shows the linear equations that correlate bifurcation angles and fitting coefficients *γ* and *η*. The fitting equations are as follows:

γ=−1.101α (11)

η=−0.005α (12)

By merging Eqs (10), (11) and (12), a new equation can obtain as follows:

$$\frac{\Delta T_{\text{r}}}{\Delta T_{op}}=0.282e^{-1.101\left(\frac{\alpha r}{H}\right)}+0.718e^{-0.005\left(\frac{\alpha r}{H}\right)}$$ (13)

However, Eq (13) only considers the effect of the bifurcation angle on longitudinal temperature decay in the branch. In addition to the bifurcation angle, the heat release also influences the longitudinal temperature decay in the branch. Therefore, the following equation can correlate the data more comprehensively.


$$\frac{\Delta T_{\text{r}}}{\Delta T_{op}}=0.282e^{\gamma \prime \left(\frac{\alpha r}{H}\right)}+0.718e^{\eta \prime \left(\frac{\alpha r}{H}\right)}$$ (14)

**Fig 13:**
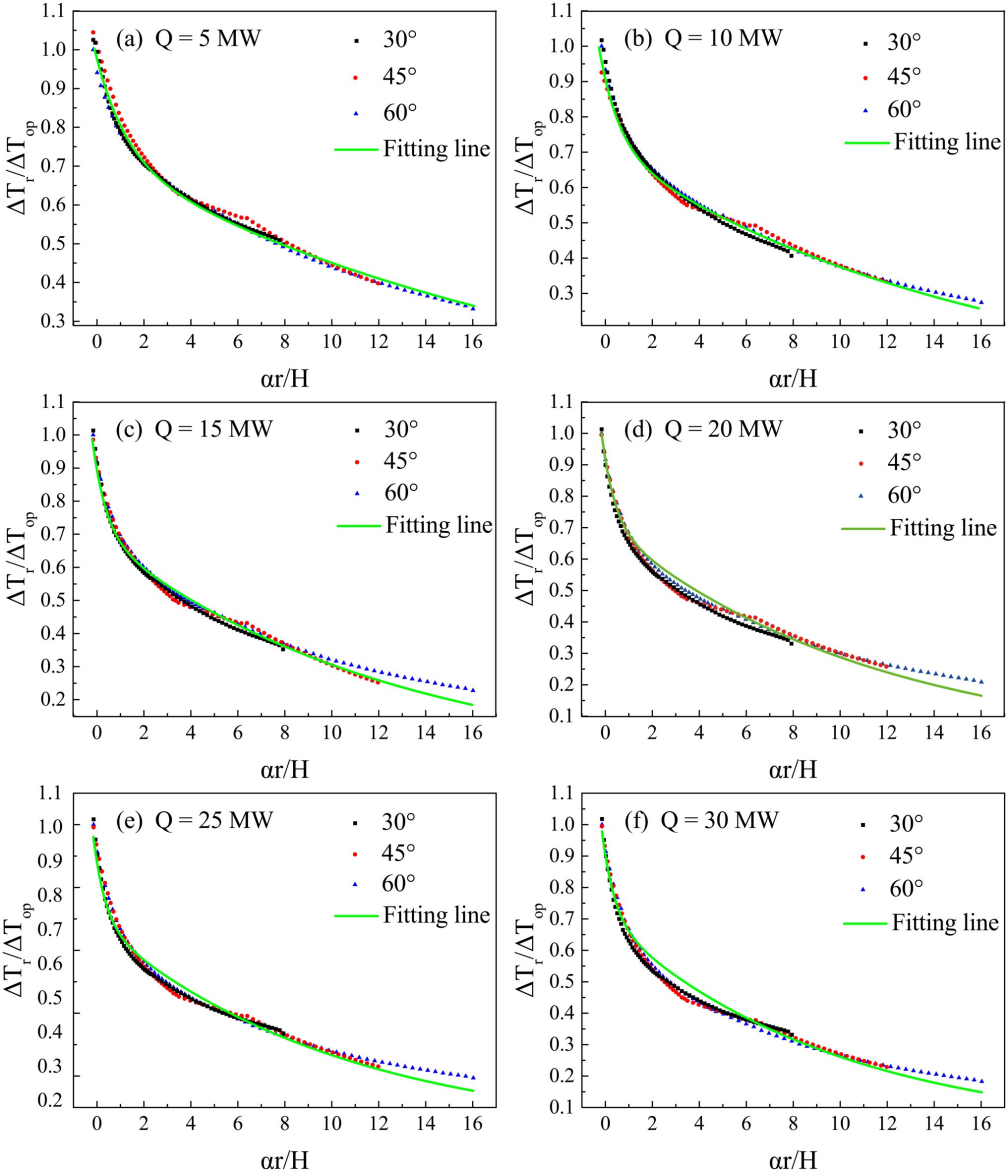
Dimensionless temperature rise (Δ*T*_r_/Δ*T*_*op*_) against new dimensionless distance (*αr*/*H*) along longitudinal direction of branch tunnel for various HRRs.

**Fig 14:**
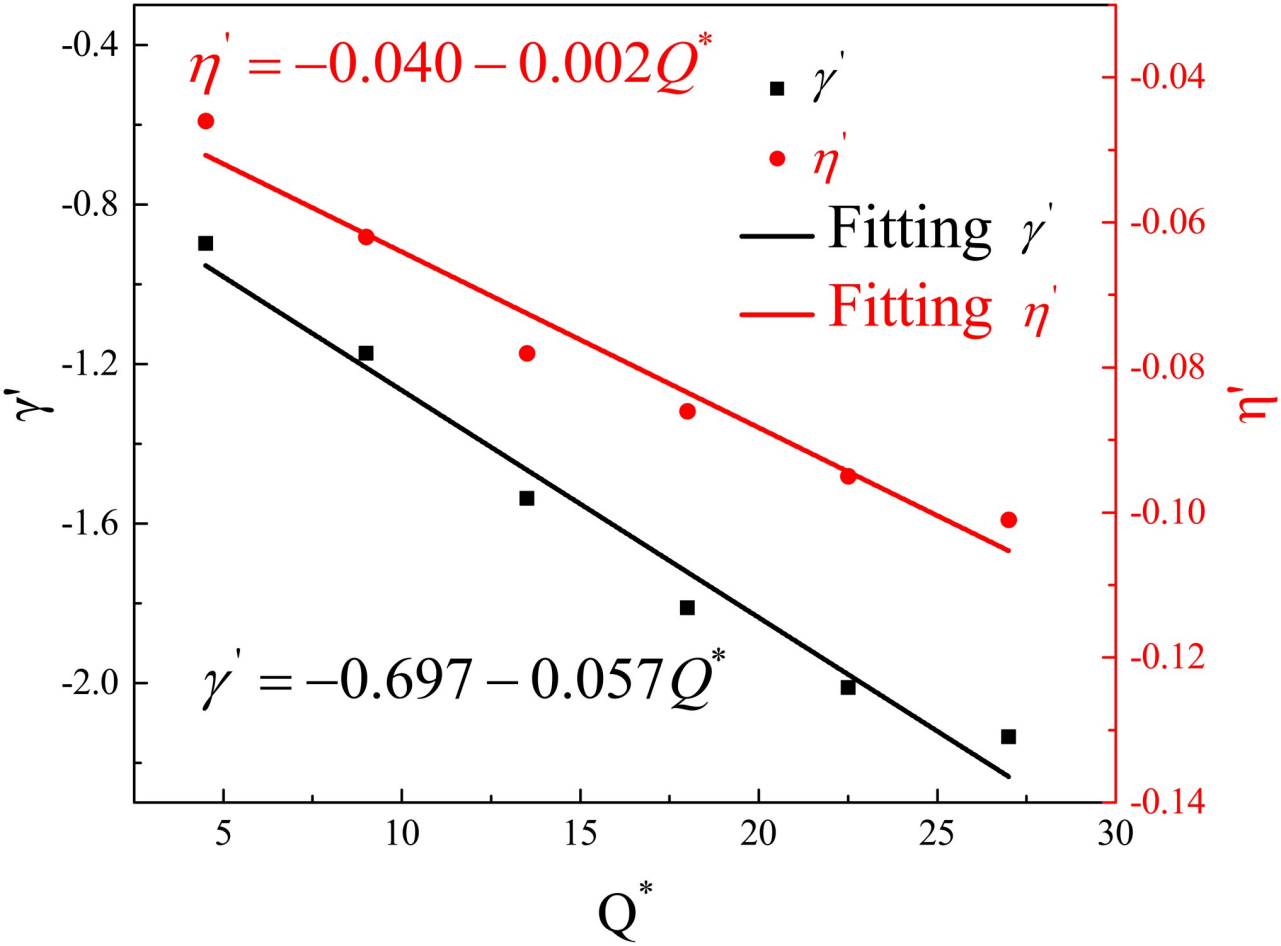
The fitting coefficients of *γ*′ and *η*′ against dimensionless heat release rate Q*.

Fig 13(a)–13(e) is Δ*T*_r_/Δ*T*_*op*_ against *αr*/*H* and the fitting results using Eq (14) for various HRRs. It shows that for various HRRs, longitudinal temperature decay in the branch tunnel follows a similar exponential equation, but coefficients of the equation are different. Table 4 lists the coefficients *γ*′ and *η*′, and Fig 14 shows these coefficients against dimensionless HRR. The following linear function can relate coefficients of *γ*′ and *η*′ with dimensionless HRR:

$$\gamma \prime =-0.697-0.057Q^{*}$$ (15)

$$\eta \prime =-0.040-0.002Q^{*}$$ (16)

**Table 4:** The values of fitting coefficients *γ*′ and *η*′.

Q*	4.5	9.0	13.5	18.0	22.5	27.0
*γ*′	‒0.898	‒1.173	‒1.537	‒1.811	‒2.011	‒2.135
*η*′	‒0.046	‒0.062	‒0.078	‒0.086	‒0.095	‒0.101

Finally, the following equation can be obtained by merging Eqs (15), (16) and (14):

$$\frac{\Delta T_{\text{r}}}{\Delta T_{op}}=0.28e^{(-0.70-0.057Q^{*})\left(\frac{\alpha r}{H}\right)}+0.72e^{(-0.04-0.002Q^{*})\left(\frac{\alpha r}{H}\right)}$$ (17)

This equation takes bifurcation angle and HRR into account simultaneously. Therefore, it can predict the longitudinal temperature decay in the branch of a bifurcation tunnel fire.

**Fig 15:**
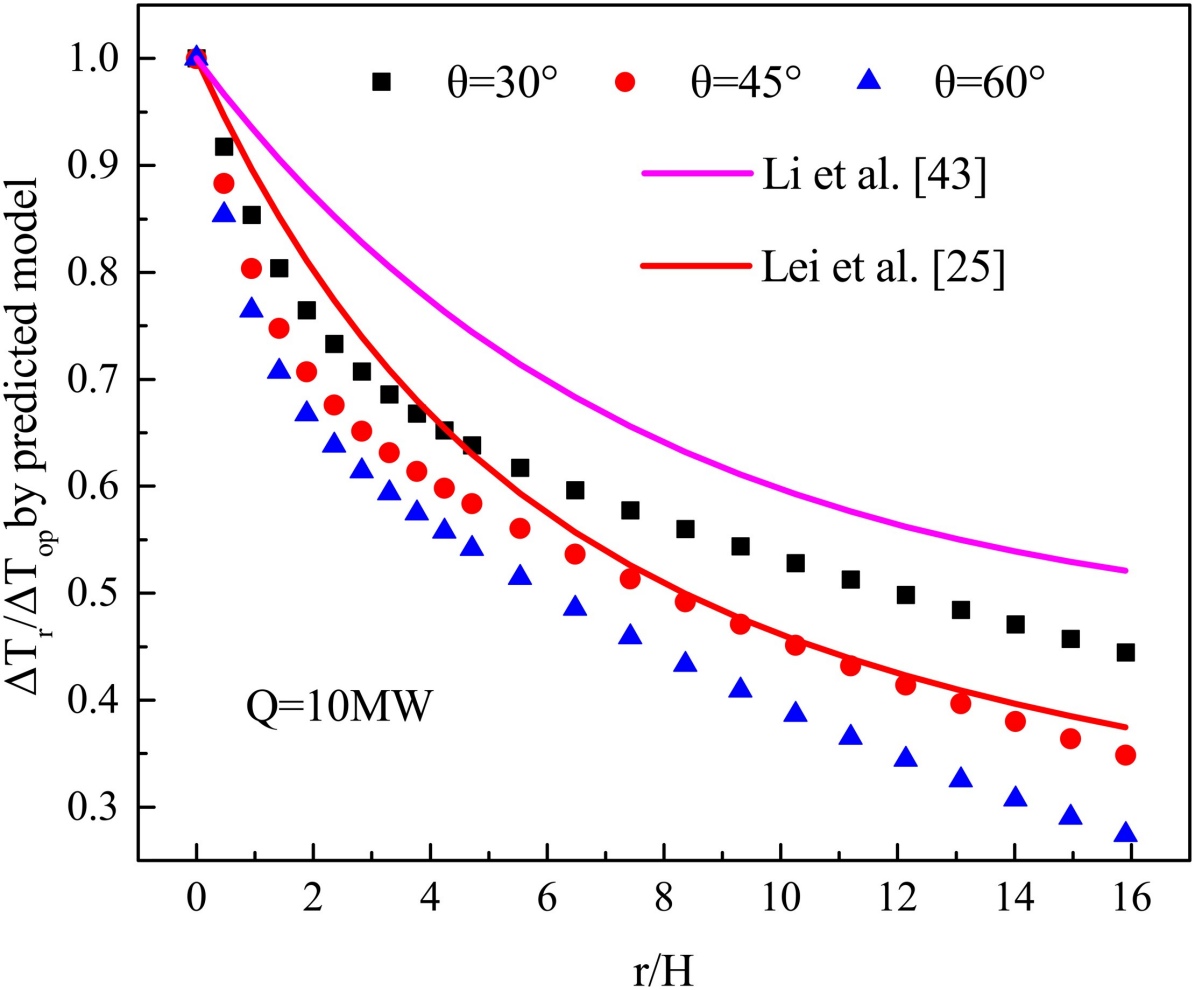
Comparisons between current research and published data [25, 43].

Fig 15 shows the values calculated by Eq (17) and the models derived from Li et al. [44] and Lei et al. [32]. The longitudinal temperature profile follows the exponential decay, although there are some differences between these studies. For example, according to the data of single tunnel fire tests, Li et al. [44] derived a double exponential formula to predict the longitudinal temperature of a tunnel fire. However, the authors did not consider the influence of the bifurcation angle on the temperature profile, so the values calculated by Li’s model are higher than values predicted by Eq (17). Furthermore, Lei’s model was derived by fire experiments in a model tunnel with a bifurcation angle of 45°. Although the bifurcation angles between Lei’s experiments and the current study are equal, the connection mode of the main tunnel and branch is different. In Lei’s experiments, the main tunnel and branch are connected by a flexible joint, but they are directly connected in the current study. Therefore, the temperature profile of Lei’s experiments and the current study have some differences because a flexible joint hinders the smoke flow and results in the smoke accumulating near the branch entrance. So, the values predicted by Lei’s model are higher than the values calculated by Eq (17). As far away from the branch entrance tunnel, the influence of connection mode on smoke gradually weakened. Therefore, the differences between the values predicted by Lei’s model and the values calculated by Eq (17) gradually decrease, and these values are in good agreement when r/H is larger than seven. However, Lei’s model did not consider different bifurcation angles on the longitudinal temperature decay of a bifurcation tunnel fire. Hence, when the bifurcation angles are 30° and 60°, the values calculated by Lei’s model are significantly different from the values calculated by Eq (17).

## 4. Conclusions

Numerical simulations were conducted to investigate the coupled effects of bifurcation angle and heat release rate on the temperature profile characteristics of a bifurcation tunnel fire under natural ventilation. The following conclusion can be drawn:

1. The maximum temperature of a bifurcation tunnel fire increases with the bifurcation angle, and its value is higher than a traditional single tunnel. Additionally, the maximum temperature position would incline upstream of the main tunnel, which is also different from a traditional single tunnel.
2. Two new equations which take the bifurcation angle and the heat release rate into account are deduced. These equations can predicate the maximum temperature under the tunnel ceiling and the longitudinal temperature decay in the branch when a fire occurs in a bifurcation tunnel.

It should be noted that the main tunnel and branch may present any cross angle in an actual bifurcation tunnel. However, the simulations in this paper only considered three bifurcation angles. Therefore, the temperature profile of bifurcation tunnel fire should be investigated by taking more bifurcation angles into account in further research. Additionally, the models proposed in this paper should be verified in further applications by full-scale experiments.
