# Expression of Concern: A study on the temperature profile of bifurcation tunnel fire under natural ventilation

**DOI:** 10.1371/journal.pone.0350792

**Published:** 2026-06-04

**Authors:** The PLOS One Editors

The *PLOS One* Editors issue this Expression of Concern for this article [1] because of concerns identified about the peer review process. Readers are advised to interpret the article [1] with caution.
